# Engineering Plant Cell Fates and Functions for Agriculture and Industry

**DOI:** 10.1021/acssynbio.4c00047

**Published:** 2024-04-04

**Authors:** Connor Tansley, Nicola J. Patron, Sarah Guiziou

**Affiliations:** † Engineering Biology, Earlham Institute, Norwich Research Park, Norwich, NR4 7UZ United Kingdom; ‡ Department of Plant Sciences, University of Cambridge, Downing Street, Cambridge, CB2 3EA United Kingdom

## Abstract

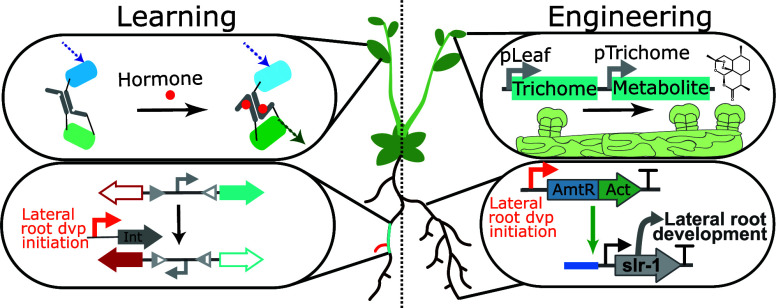

Many plant species are grown to enable access to specific organs or tissues, such as seeds, fruits, or stems. In some cases, a value is associated with a molecule that accumulates in a single type of cell. Domestication and subsequent breeding have often increased the yields of these target products by increasing the size, number, and quality of harvested organs and tissues but also via changes to overall plant growth architecture to suit large-scale cultivation. Many of the mutations that underlie these changes have been identified in key regulators of cellular identity and function. As key determinants of yield, these regulators are key targets for synthetic biology approaches to engineer new forms and functions. However, our understanding of many plant developmental programs and cell-type specific functions is still incomplete. In this Perspective, we discuss how advances in cellular genomics together with synthetic biology tools such as biosensors and DNA-recording devices are advancing our understanding of cell-specific programs and cell fates. We then discuss advances and emerging opportunities for cell-type-specific engineering to optimize plant morphology, responses to the environment, and the production of valuable compounds.

## Introduction

**Figure 1:**
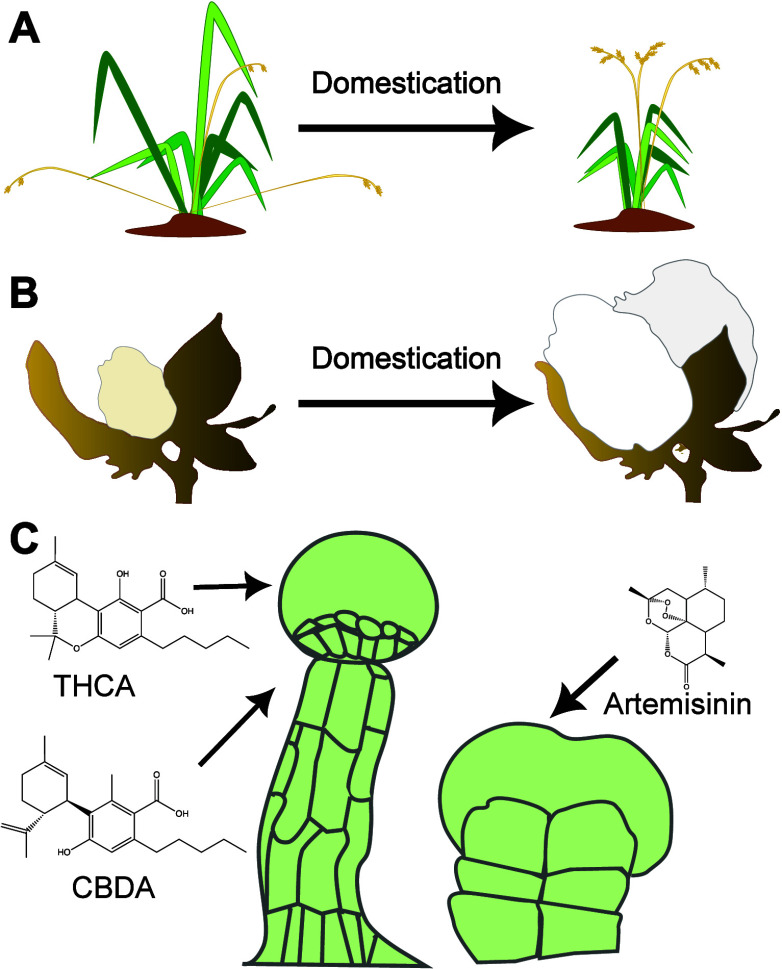
Many plants are cultivated for specific organs, tissues, or cells, which have been targets for selective breeding. A. Changes in inflorescence size and architecture and a compact plant morphology contributed to yield increases in both *Oryza glaberrima* (African rice) and *Oryza sativa* (Asian rice). B. The domestication of *Gossypium hirsutum* (cotton) increased the length and number of seed coat hairs as well as the ease of detachment. C. Many plant natural products are found in trichomes, including artemisinin found in the leaf glandular trichomes of *Artemisia annua* (sweet wormwood) (right), and tetrahydrocannabinolic acid (THCA) found in the stalked glandular trichomes of female *Cannabis sativa* L (cannabis) flowers (left). Increasing the trichome density has been used as a strategy to increase product yields.

Plant cultivation is considered to be one of the most important cultural transitions in human history. Today, 40% of land mass is dedicated to growing plants for food as well as for products such as wood, textiles, dyes, and medicines.^1^ For many species, the value is associated with a specific organ, tissue, or cell-type (Figure 1). In food production, this is exemplified by seeds harvested from grain crops and roots from *Daucus carota* subsp. *Sativus* (carrot), tubers from *Solanum tuberosum* (potato), stems from *Apium graveolens* (celery), and the fruit of numerous species, including *Solanum lycopersicum* (tomato). Industrial products include seed hairs from *Gossypium hirsutum* (cotton), extraxylary fibers from *Linum usitatissimum* (flax), and natural products from numerous species that often accumulate in specialized cell types. Although recent efforts have sought to maximize the use of plant biomass, for example by producing biofuels from *Zea mays* (corn) leaves and chippings for playgrounds and gardens from timber waste, efforts are still primarily focused on maximizing yields of target organs and products.

Selective breeding has improved crop yields by increasing the size, number, and quality of valuable harvested organs or tissues and by modifying plant architecture, optimizing it for large-scale cultivation (Figure 1A). For example, domestication of *Oryza sativa* (rice) fixed semidwarfism and increased inflorescence branching to prevent lodging and increase grain-yield, respectively.^2^ Similarly, tomato breeding fixed a more compact morphology with more fruit per stem,^3^ while cotton breeding selected for longer and more plentiful seed coat hairs to produce larger bolls^4^ (Figure 1B). Many of the mutations that underlie these phenotypes have been identified to be key regulators of cellular identity. For example the compact morphology of tomato is attributed to a mutation in *SELF-PRUNING* (*SELF*), which regulates the development of terminal flowers instead of further branching stems.^5^ Fruit yield per stem is attributed to a mutation in *WUSCHEL HOMEOBOX9* (*WOX9)*, a homeobox gene involved in maintaining the shoot’s capacity for growth.^6^ The cell-type-specific expression of such regulators therefore controls the shape, size, arrangement, and abundance of plant organs.

In species cultivated for compounds used in health care and industry, the location of the target product is often even more limited, accumulating in rare and specialized cell types. Some cell types, such as trichomes (epidermal outgrowths), are relatively easy to isolate and characterize, and many natural products have been found to be produced by these cells. For example, the antimalarial artemisinin, from *Artemisia annua* (sweet wormwood) and tetrahydrocannabinolic acid (THCA) from *Cannabis sativa* (cannabis) accumulate in leaf and floral glandular trichomes, respectively^7,8^ (Figure 1C). Other specialized plant cells are relatively poorly studied as they are embedded within complex tissues, but recent advances in single-cell technologies are producing novel insights. For example, in *Catharanthus roseus* (Madagascar periwinkle), the biosynthesis of vindoline, a precursor of the anticancer drugs vinblastine and vincristine, was located to laticifer and idioblast cells.^9^ However, relatively little is known about the differentiation and development of these cell types.^10^

Synthetic biology provides the potential to reprogram plant cell fates to engineer novel architectures and manipulate cellular identities. It could be applied to further optimize the architecture of cultivated species to, for example, engineer forms better suited to the water and nutrient stress events that are predicted to be more frequent and severe in a changing climate.^11^ Synthetic biology is already revolutionizing access to plant natural products via pathway reconstruction in heterologous hosts.^12^ However, extraction from field-grown crops or cultured tissue continues to be the most economical route to many compounds. The ability to engineer cell identities and conduct cell-type-specific metabolic engineering in the native host provides the potential to increase the yield of high-value compounds. Achieving these aims requires detailed information about plant cell-identities and of the cellular events that underlie cell fate. In this Perspective, we first discuss how synthetic biology can contribute to deepening our understanding of plant cell-types and cell-specific programs. We then discuss opportunities for engineering cell fates and functions for the benefit of agriculture and industry.

## Synthetic Biology Approaches for Elucidating Plant Cellular Functions and Lineages

In the past, many key regulators of cellular identity were discovered either in mutation screens or using microarrays and RNA-sequencing to identify genes that change expression in specific organs or tissues. This was followed by functional characterization of candidates by overexpression/silencing and promoter-fusions to glucuronidase, luciferase, or fluorescent reporters (recently reviewed in refs (13 and 14)). Later, the identification of genes expressed in specific cells was enabled by microdissection, isolation of marked nuclei, and fluorescence-activated cell sorting (FACS) of protoplasts isolated from reporter lines.^15^ In recent years, single-cell omics of protoplasts and isolated nuclei has led to an explosion in data. However, the identification and comparison of cell types across the diversity of plant lineages from gene-expression signatures remains a significant challenge.^16^ The advent of spatial genomics, which allows high-throughput fluorescent *in situ* hybridization (FISH) is beginning to address this, though to date it has only been applied to a few plant species.^17,18^ To understand how metabolic pathways are organized across cell-types, single cell transcriptomics and metabolomics have been integrated to link gene expression with the presence of specific compounds.^19^

Omics techniques provide a snapshot of the molecular state of cells but are destructive, making it challenging to observe dynamics. In contrast biosensors enable the high-resolution monitoring and quantification of metabolites, nutrients, hormones, small molecules, and gene expression within individual cells while preserving spatial information.^20^ In recent years, the ability to design novel biosensors and tune their function has been revolutionized by synthetic biology, resulting in an expansion of the biosensors for plants.

**Figure 2:**
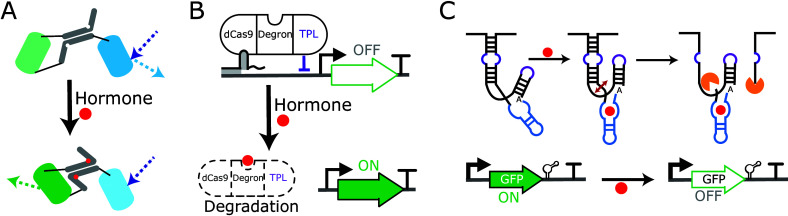
Examples of biosensors deployed in plants A. FRET biosensors for hormone detection.^21^ In the absence of the hormone, excitation results in emission of the blue fluorophore. The presence of the hormone enables energy transfer between the two fluorophores, leading to the emission from the green fluorophore. B. HACR (Hormone Activated Cas9 Repressor) biosensors are composed of dCas9 fused to hormone-activated degron, and transcriptional repressor (topless; TPL) domains.^26^ In the absence of hormone, the HACR associates with its sgRNA, binding to and repressing the promoter of the output/reporter module. In the presence of the hormone, the HACR is degraded, leading to derepression. C. A riboswitch biosensor for theophylline.^28^ The RNA aptamer is encoded in the 3′ untranslated region of the output/reporter module (bottom). In the absence of theophylline, the aptamer conformation occludes the RNA cleavage site and results in translation (top-left). In the presence of theophylline (red), a conformational change exposes the cleavage site, leading to RNA-degradation (top-right).

Direct biosensors, consisting of a single multifunctional module, are typically based on fluorescent proteins with optical properties dependent on the signal (e.g., pH, Ca^2+^), or are a fusion of two Forster Resonance Energy Transfer (FRET)-compatible fluorophores and a ligand-binding domain. In plants, direct biosensors have been designed to detect phytohormones including auxin^21^ (Figure 2A) and ABA,^22^ nutrients such as inorganic phosphate,^23^ and stress, e.g., via pH.^24^ Indirect biosensors have separate sensing, processing, and output components, which decouples sensing from output. Examples deployed in plants include those based on transcriptional regulation, e.g., a copper sensor fused with a Gal4 activator;^25^ on post-translational modification, e.g., a dCas9-based biosensor with a degron for the detection of plant hormones^26^ (Figure 2B); and on translation regulation, e.g., riboswitch-based biosensors for the detection of thiamine^27^ and theophylline^28^ (Figure 2C). Modularity of detection and output responses was demonstrated using a plant hormone receptor (PYR1) as a reprogrammable scaffold for detecting a range of molecules including cannabinoids and organophosphates, combined with various ligand-responsive outputs.^29^

**Figure 3:**
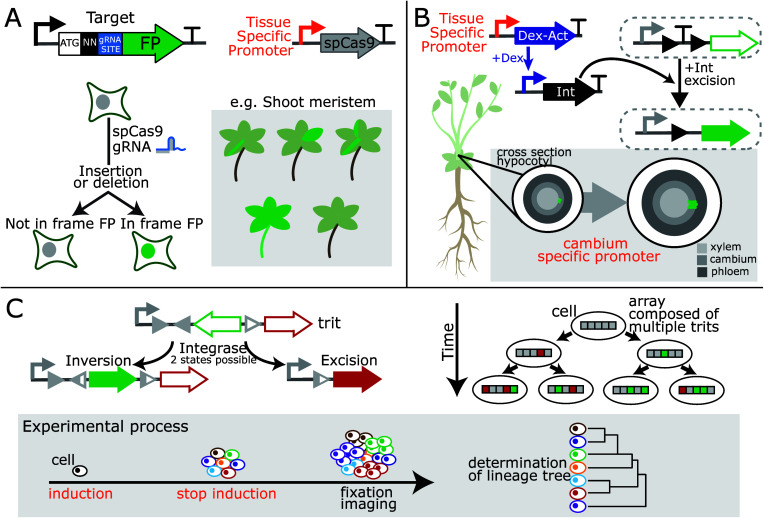
DNA-recording devices create heritable memory, allowing cell lineages to be traced. A. A CRISPR-Cas9-based DNA-recording device^30^ composed of an out-of-frame fluorescent protein (FP) with a gRNA target site at the 5′ extremity of the coding sequence. The promoter controlling Cas9 expression is activated by molecular signals (transcription factors) in progenitor cells, leading to insertion/deletion events, some of which restore the reporter reading frame, enabling the detection of daughter cells by sequencing or FP visualization. B. An integrase-based DNA-recorder for analysis of cambium stem cells.^32^ Expression of an integrase (Cre) is activated in cambium stem cells in the presence of dexamethasone (DEX). The integrase mediates the excision of the terminator (T) in the output module, leading to the expression of the FP. C. Integrase-editable memory by engineered mutagenesis with optical *in situ* readout (intMEMOIR).^33^ Implemented in mammalian cell cultures, this method uses three-state memory elements (trits) in which no editing = no expression; inversion = expression of barcode 1; and excision = expression of barcode 2. Multiple occurrences of this unit are present in each cell, and their state is read using smFISH. After the induction of integrase expression, DNA editing will accumulate over time, and cell lineages can be recapitulated by analyzing the state of these multiple units. This enables the retracing of the cell-lineage trees.

In general, live imaging is required to detect signals from *in vivo* biosensors. However, live imaging of large species is challenging, and the autofluorescence of many plant tissues can be difficult to overcome. An alternative is to record biosensor signals into DNA. This enables the presence of even transient signals to be detected after their presence either by DNA-sequencing or by imaging a fluorescent reporter, the expression of which is activated/repressed by biosensor-induced genetic changes. An advantage of these so-called DNA-recording devices is that the memory of detection is heritable, allowing cell lines to be traced. For this reason, they have been applied to understanding cell fates, cell lineages, and organ development. For example, CRISPR-Cas9-based DNA-recording was used to track cellular lineages in *Arabidopsis thaliana* (*Arabidopsis*) and *Marchantia polymorpha*(30) (Figure 3A). Integrase-based DNA-recorders were implemented in *Arabidopsis* to detect the expression of transcription factors that control lateral root development,^31^ and for analysis of cambium stem cells^32^ (Figure 3B). As plant cells are not generally mobile, live imaging has been used to track some cell lineages. Nevertheless, these new techniques provide an opportunity to understand the molecular mechanism of cellular transitions and can be used to differentiate lineages over different time periods while retaining spatial information. They also provide new opportunities for tissues and species for which live-imaging has presented technical challenges. In animals, these approaches are more advanced and have been used to obtain complete maps of cell ancestry for tissues and organs by multiplexing DNA editing sites with different probabilities forming molecular barcodes^33,34^ (Figure 3C). The barcodes can be either read using single-cell sequencing to reconstitute the cell-lineage tree or visualized by single molecule fluorescence *in situ* hybridization (smFISH). The application of such methods to plants might enable a complete map of cell ancestry and could be combined with omics methods to link cell lineages to cell fates and molecular states of cells.

## Emerging Technologies for Controlling Plant Development and Performance

**Figure 4:**
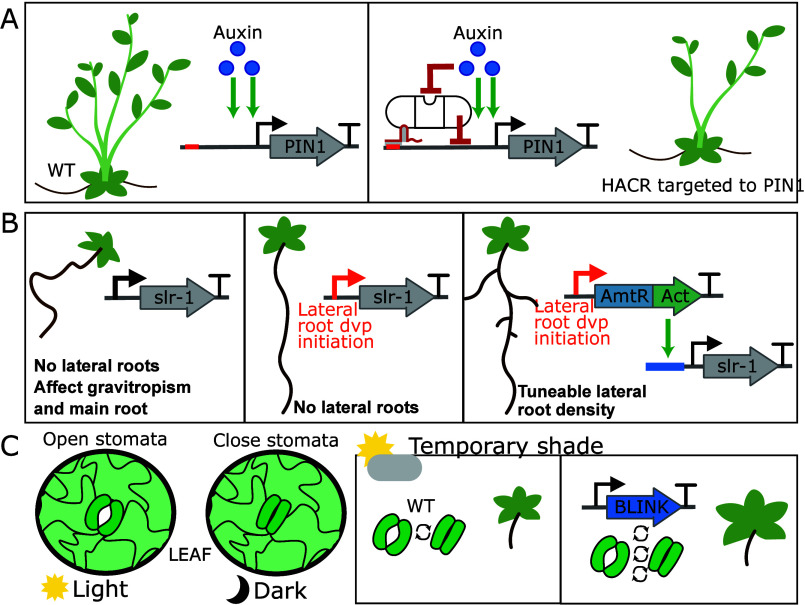
Engineering plant development and performance. A. Auxin-repression of axillary bud development is dependent on the auxin-mediated activation of the auxin transporter PIN-FORMED1 (PIN1). A synthetic hormone-activated Cas9-based repressor (HACR) decreased the activation of expression of PIN1 by auxin, reducing feedback and leading to fewer side branches.^26^ B. Expression of a gain-of-function mutation in the developmental regulator *INDOLE-3-ACETIC ACID INDUCIBLE 14* (*IAA14*) called *solitary root* (*slr-1*) eliminates root branching but also hinders root gravitropism, root hair development, and primary root growth (left). Lateral-root-stem-cell-specific expression (center) restores gravitropism, root hair development, and primary root growth. Expression of *slr-1* (right) was tuned by controlling expression via cell-type-specific expression of a synthetic transcription factor (AmtR-VP16) and a cognate synthetic promoter with one, two, four, and six copies of the AmtR operator, enabling control over branching.^37^ C. Leaf stomata open in light and close in the dark to limit water-loss (left). To accelerate stomatal kinetics, a synthetic potassium channel fused to a blue-light-triggered photoreceptor (BLINK1) was expressed from a guard cell-specific promoter.^38^ This increased growth under fluctuating white light mimics the temporary shade conditions experienced by passing clouds or shadows.

The architecture of plants is most often defined as the three-dimensional organization of the major organs. This can influence organ quantity, location, and size, which are significant contributors to the yields of target products. In aerial tissues, architecture includes the branching pattern of the stem(s) as well as the size, shape, and position of leaves and inflorescences. Fewer side branches are often beneficial in field systems, as they allow planting at higher density and facilitate mechanized harvesting. Compact architectures suited to high-density cultivation are also desirable for vertical farms, which require less water and can increase yield per unit area of land.^35^ Synthetic biology can be used to engineer these traits. For example, in some species, auxin prevents the development of axillary buds via a complex mechanism dependent on the strength of repression of auxin transporter PIN-FORMED1 (PIN1). Khakhar and colleagues engineered an auxin-activated HACR (hormone-activated Cas9-based repressors) to decrease the activation of expression of PIN1 by auxin and reduce feedback, leading to fewer side branches^26^ (Figure 4A).

In root systems, the number, position, and density of lateral roots are essential parameters for optimum nutrient and water absorption.^36^ Synthetic biology approaches have been used to tune root architecture, including the combination of cell-type-specific expression with synthetic signal processing. When expressed via the native promoter, a mutant allele of a developmental regulator, solitary root (*slr-1*), affects root branching, gravitropism, root hair development, and primary root growth. Brophy and colleagues used buffer gates to tune the location and level of *slr-1* in order to control root branching and limit unwanted effects^37^ (Figure 4B).

Plants also need to adapt to changes in growth conditions, including fluctuating light conditions. Stomata, pores in the leaf epidermis that regulate gas exchange, are generally open in light to enable CO_2_ absorption but close in the dark to limit water loss via transpiration. To accelerate stomatal kinetics, reducing the time in which photosynthesis is repressed, researchers engineered a optogenetic system consisting of a synthetic potassium channel and a blue-light sensor (BLINK1) under the control of guard cell-specific promoter^38^ (Figure 4C). Implementation resulted in increased growth under fluctuating white light without increasing water consumption.

Finally, certain cell types, architectures, and organs are present only in specific plant lineages. For example, symbiotic nitrogen fixation in the nodules of legumes is beneficial for plant growth. Many researchers are exploring engineering strategies to introduce nitrogen-fixation or symbiont-signaling symbiosis into nonlegumes (for a review, see ref (39)), but it remains challenging. Similarly, efficient photosynthesis at high temperatures is enabled by the specific leaf cellular architecture and photosynthetic biochemistry of C4 species, such as corn. Engineering the internal structure of the leaves of C3 plants such as rice is being explored to improve photosynthetic capacity (for a review, see ref (40)). In both of these cases, the development of strategies to engineer cellular identities and tissue architectures is likely to be beneficial.

## Toward Cell-Type-Specific Metabolic Engineering

**Figure 5:**
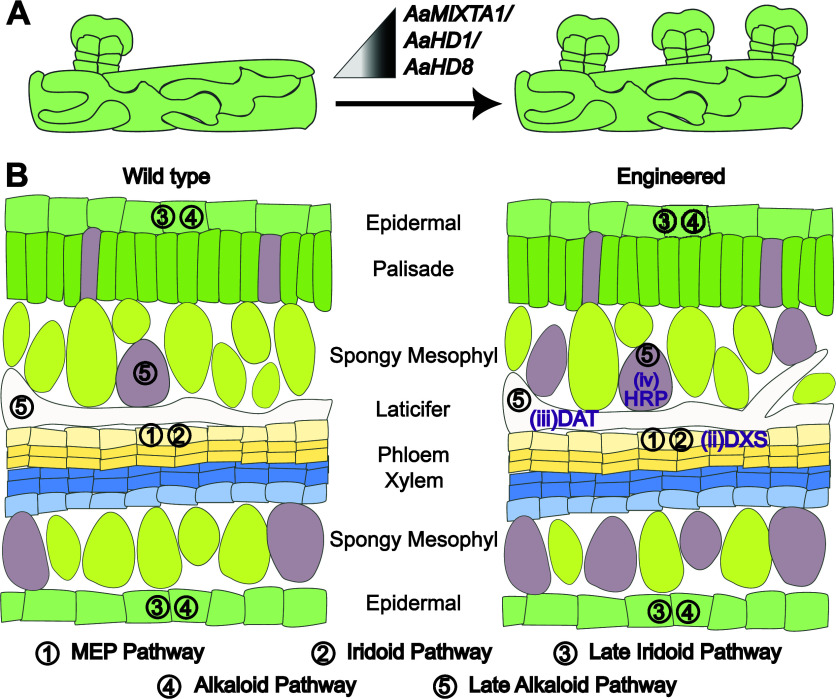
Strategies for increasing natural-product yields via cell-type-specific engineering. A. Increased densities of glandular secretory trichomes in *A. annua* via network engineering to tune the abundance of developmental regulators AaMIXTA1 (*MIXTA-Like 1*), AaHD1 (*HD-ZIP IV 1*), and AaHD8 (*HD-ZIP IV 8*). B. The vinblastine biosynthetic pathway is divided into five subpathways, spatially separated across four cell types in *Catharanthus roseus* (left).^19^ The MEP pathway (1) and Iridoid pathway (2) are predominantly in phloem-associated parenchyma cells. The late iridoid pathway (3) and alkaloid pathway are in epidermal cells, and the late alkaloid pathway (5) in idioblast cells (brown) and laticifer cells. Yield increases (right, purple annotations) might be achieved via (i) developmental reprogramming to increase laticifer branching or idioblast density, (ii) upregulation of 1-deoxy-d-xylulose 5-phosphate synthase (DXS) in phloem-associated parenchyma, (iii) upregulation of the rate-limiting acetyl-CoA:4-*O*-deacetylvindoline 4-*O*-acetyltransferase (DAT) in idioblast and laticifer cells, and (iv) expression of horseradish peroxidase in idioblast or laticifers to aid oxidative coupling of catharanthine and vindoline.^19^

As noted above, many valuable bioactive and industrially relevant compounds are found in plants, often accumulating in specific tissues and cells. These include trichomes and laticifer cells in vascular plants^7,8,41^ and oil bodies in liverworts.^42^ This may be linked to specific functions, or because biosynthesis presents metabolic challenges to other cells such as toxicity and competition for precursors.^43,44^ Increasing the number of specialized cell-types represents a strategy to increase product yields. As several trichome-associated compounds have been associated with defense, this could also provide a route to improved resistance to pests and pathogens. Cannabis breeding has manipulated the number and type of trichomes to improve yields of THCA.^45^ In sweet wormwood, glandular trichome density was increased by manipulating the levels of developmental regulators, improving artemisinin yields (for a review, see ref (46)) (Figure 5A). Equivalent approaches might be used to increase the population of idioblast or laticifer cells, which are also known to accumulate valuable natural products in other species, including Madagascar periwinkle.^9^ However, relatively little is known about the developmental programs that lead to the differentiation of these cell-types.^47^

A second strategy is to upregulate production by cell-type-specific metabolic engineering. Strong, constitutive promoters can negatively affect plant growth and development.^48^ Further, increasing precursor availability in a cell-type-dependent manner rather than constitutively would reduce the transcriptional burden of the synthetic circuits, the impacts of which have been noted in other systems.^49,50^ Previously, it was found that overexpression of acetyl-CoA:4-*O*-deacetylvindoline 4-*O*-acetyltransferase (DAT), a rate-limiting enzyme in terpene-indole alkaloid biosynthesis in Madagascar periwinkle, led to increased vindoline biosynthesis.^51^ Recent single-cell data suggest that, as its precursor is mainly available in idioblast and laticifer cells, upregulation in these cell types may be sufficient (Figure 5B). Cell-type-specific engineering is likely to be even more beneficial when engineering precursor availability for the first committed steps. Enabling this in specific cells would avoid metabolic burdens in cells that lack the downstream pathway. In Madagascar periwinkle, precursor availability could be specifically engineered in leaf epidermal cells (Figure 5B). Similarly, it has been proposed that the rate-limiting steps for production of vinblastine is the oxidative coupling of catharanthine and vindoline, which may be improved by the expression of a peroxidase in idioblast cells^19^ (Figure 5B).

An alternative route to accessing natural products is heterologous biosynthesis. Although microbial production chassis are widely used, pathway reconstruction in plants, particularly species of *Nicotiana* including tobacco *(N. tabacum)* and an Australian relative, *N. benthamiana*, have been successful.^52^ To date, most studies have expressed pathways using strong constitutive promoters. This is generally successful when expression is transient, achieved by the leaf-infiltration of multiple strains of the *Agrobacterium* shuttle chassis each carrying individual genes.^53^ However, as this strategy requires containment glasshouses and bacterial cultivation, it may be economically viable only for high-value products. In contrast, field production of recombinant products in stable transgenics, e.g., *N. tabacum*, is low-cost,^54^ but requires the stable integration of synthetic pathways into the nuclear or plastid genome. Here, strong, constitutive promoters can lead to detrimental phenotypes, including impaired growth.^48^ Engineering strategies that limit expression to specific organs such as fruits, specific cell types, or tightly inducible expression are less likely to impact growth. A novel application for metabolically engineered plants is as living dispensers of volatile chemicals for pest control.^55^ In this case, production in trichomes and increases in trichome density are expected to be beneficial for product release.

Other plant species proposed for photosynthesis-driven bioproduction include the moss *Physcomitrium patens*(56) and the liverwort *M. polymorpha*.^57^ The latter produces complex oil bodies, specialized organelles that accumulate unique compounds including bisbibenzyls and several isoprenoid-derived compounds.^58^ It has been demonstrated that overexpression of the transcription factor, *MpERF13*, increases the number of oil body cells, which could improve its utility as a production chassis, but has deleterious effects on growth.^59^ These negative impacts might be avoided by precise spatiotemporal control of the expression.

## Conclusions

Synthetic biology approaches have been applied to engineer a number of model plant species, demonstrating their utility for elucidating and engineering developmental programs and for metabolic engineering. While such proof-of-concepts are important, there is now a need to accelerate the engineering of crop plants for agriculture. The recent, rapid uptake of single-cell omics by the plant community is already producing a wealth of information about cellular functions in a wide range of agriculturally and industrially important species.^16^ Advances in synthetic biosensors and DNA-recording devices (Figure 2 and 3) are also likely to provide novel insights into the molecular events that lead to cell differentiation and cell-type-specific functions. Currently, the plant synthetic biology community experiences bottlenecks imposed by low-efficiency and laborious plant transformation protocols. Methods to study cellular programs might also be applied to better understand and engineer improvements in plant regeneration. Ultimately, these advances will inform cell-type-specific engineering strategies to optimize plant body plans and accelerate the production of plant varieties for a rapidly changing climate and for the sustainable production of compounds for health and industry.
